# A framework of psychological compensation in attention deficit hyperactivity disorder

**DOI:** 10.3389/fpsyg.2015.01580

**Published:** 2015-10-28

**Authors:** Julia Merkt, Tilman Reinelt, Franz Petermann

**Affiliations:** Center of Clinical Psychology and Rehabilitation, University of BremenBremen, Germany

**Keywords:** ADHD, compensation, heterogeneity, diagnosis, recovery, development

## Abstract

The term compensation is widely used in the context of attention deficit hyperactivity disorder (ADHD), yet, it is neither defined nor theory driven. Adapting a model of psychological compensation (Bäckman and Dixon, 1992) to fit ADHD research is the aim of this review: we will (1) introduce the existing theoretical framework of psychological compensation, (2) discuss its applicability to ADHD and adapt the model to fit ADHD research, and (3) set up requirements for research on psychological compensation in ADHD. According to the framework psychological compensation can be inferred if a deficit (i.e., a mismatch between skill and environmental demand) is counterbalanced by the investment of more effort, the utilization of latent skills, or the acquisition of new skills. The framework has to be adapted because ADHD deficits are developmental and in individuals with ADHD compensation can appear independent of awareness of the deficit. A better understanding of psychological compensation in ADHD could foster diagnosis and interventions. Therefore, we suggest that future studies should follow a research design incorporating independent measures of deficit, compensation, and outcome as well as include individuals who compensate for their ADHD related deficits.

However, most studies of psychological compensation generate hypotheses instead of testing them. Only few of these studies are theory driven or systematically investigate compensation. Instead, compensation is often used *post hoc* in order to discuss unexpected results (Merkt et al., 2013; Merkt and Gawrilow, 2014). A true test of compensation, however, would require an independent measurement of deficits, potential compensatory mechanisms, and outcomes. Hence, on the basis of the literature it is impossible to draw conclusions about compensation in children, adolescents, or adults with ADHD. In fact, it is unclear how and when compensation for deficits, which are typical in individuals with ADHD, might occur. Therefore, in order to integrate the results of studies in children, adolescents, and adults with ADHD and to answer the question whether they compensate and how, a framework is required. In the following sections, we introduce a framework of psychological compensation (Bäckman and Dixon, 1992) and adapt it to fit ADHD research. Our aim is to develop a framework and to suggest consequential research designs to test for deficit and compensation in ADHD. Although we use examples to illustrate our arguments (Halperin and Schulz, 2006; Gormley et al., 2015), we do not attempt to fill the framework with content to answer the question whether and how individuals with ADHD compensate.

## Framework of Psychological Compensation

**FIGURE 1:**
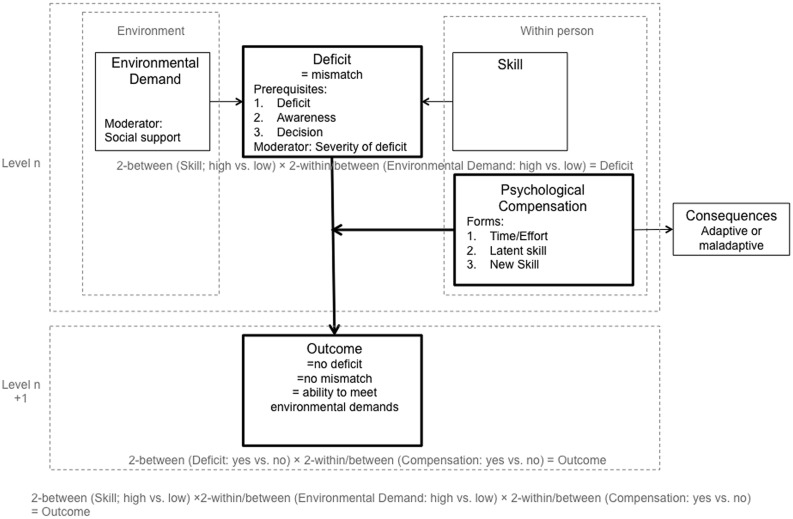
**The theoretical framework of psychological compensation**.

The theoretical framework of psychological compensation (Bäckman and Dixon, 1992) has been developed predominantly on the basis of studies investigating sensory handicaps and aging. According to the model (**Figure 1**) there are three prerequisites of compensation. The first prerequisite is defined by a deficit or mismatch between skill and environmental demand. This occurring mismatch could either be caused by an intraindividual decline in skill (e.g., during aging) or by increasing environmental demands (e.g., a child entering school). The second prerequisite states that an individual is aware of the mismatch; and the third prerequisite requires that an individual decides to compensate. If those prerequisites for compensation are fulfilled, the mismatch between skill and environmental demand can be counterbalanced by either the investment of more time/effort (drawing on normal skills), or the utilization of latent (but normally inactive) skills, or the acquisition of new skills. This process of psychological compensation can be adaptive or maladaptive, as it might not only lead to a reduction of the mismatch between skill and environmental demand but to other consequences as well (e.g., investment of more time and effort to study leads to fewer friendships). Note, that according to this definition social support or seeking a more supportive environment is not regarded as psychological compensation, since both change (i.e., reduce) the environmental demand and thus directly affect the deficit (mismatch between skill and environmental demand).

## Adaptations of the Framework to fit ADHD

### Deficit

The first prerequisite of psychological compensation is the existence of a deficit that represents a mismatch between skill and environmental demand. As the impairment criterion (American Psychiatric Association, 2013) has to be met, there has to be a deficit in individuals with diagnosed ADHD. However, in disagreement with the model of Bäckman and Dixon (1992), it is inappropriate to conceptualize the deficit in ADHD as intraindividual decline in skill or purely as a consequence of rising environmental demand. Instead, the deficit only appears if the skill is impaired or the environmental demand is high (interaction of skill and environmental demand). This is supported by theoretical assumptions and experimental findings which demonstrate that deficits in children with ADHD as compared to children without ADHD are only apparent under certain experimental conditions (Sergeant, 2005; Söderlund et al., 2010). Yet, in studies of compensation in ADHD the deficit is usually conceptualized as low level of skill, while environmental demands are rarely measured or even manipulated. The implicit assumption underlying these studies is that every sample with ADHD shows a general deficit in skills and across environments as compared to a non-clinical control sample. However, to study compensation, we need to measure the deficit as an independent variable and be specific with regard to which skill and which environment we are referring to.

**FIGURE 2:**
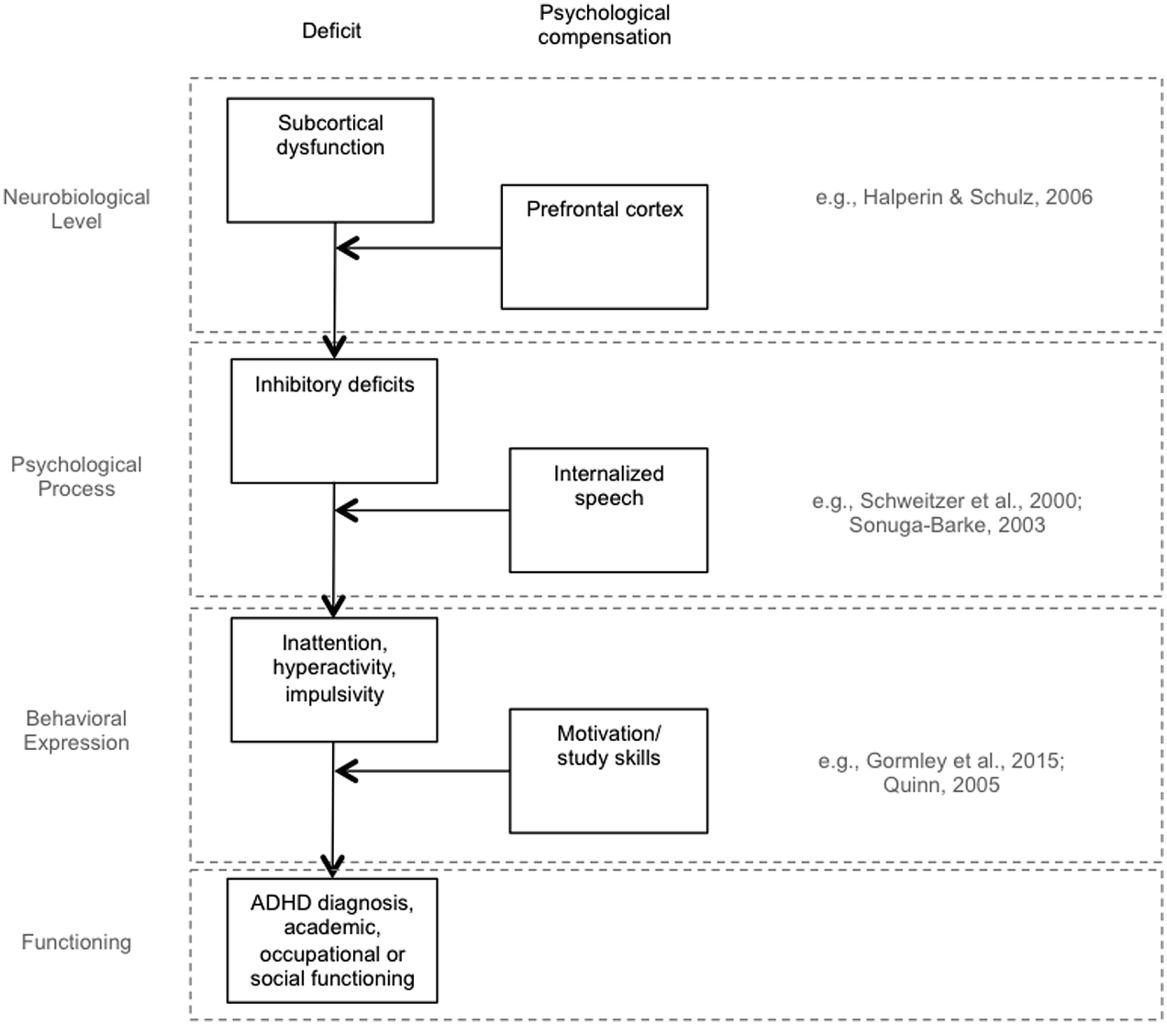
**Exemplary levels of conceptualization of psychological compensation**.

The specificity in terms of the deficit (skill and environment) we are referring to is important, as deficits in individuals with ADHD have been conceptualized at different levels (**Figure 2**): at a neurobiological level (e.g., subcortical dysfunction; Halperin and Schulz, 2006), at the level of psychological processes (e.g., inhibitory deficits, Schweitzer et al., 2000; Sonuga-Barke, 2003), at the level of behavioral expression (e.g., ADHD symptoms, i.e., less attention, less impulse and motor control; Gormley et al., 2015), and at the level of functioning (i.e., less academic or occupational achievement). This is pivotal because psychological compensation is usually conceptualized at the same level as the deficit, but the outcome is measured at another level. Compensation conceals deficits at another level of measurement. For example, if compensation by the prefrontal cortex is successful, an individual will not show ADHD symptoms anymore, although a deficit in subcortical functions remains (Halperin and Schulz, 2006). Another example is college students with ADHD: if compensation by study skills is successful there is no influence on grades, although the deficit in terms of ADHD symptoms remains (Gormley et al., 2015).

The first form of compensation, the investment of more time and effort, is problematic for research designs, because both deficit and effort are required to be measured as two independent variables. If the deficit (e.g., subcortical dysfunction) and the compensation by more effort (e.g., higher activation in the subcortical region) are measured by the same variable (e.g., activation in the subcortical region) it is impossible to draw conclusion about which of both is related to the outcome (e.g., ADHD symptoms). The second form of compensation, the utilization of latent but inactive skills is for instance shown when a student compensates for the ADHD symptoms (deficit) by high motivation (compensation) to avoid academic failure (outcome) (Gormley et al., 2015). The third form of compensation, the acquisition of new skills, matches the assumption by Halperin and Schulz (2006) that subcortical dysfunctions (deficit) remain across development but are compensated for by the development of the prefrontal cortex (compensation) leading to the recovery from ADHD symptoms (outcome). Another example of the acquisition of new skills is the development of study skills (compensation) by students with ADHD to compensate for their ADHD symptoms (deficit) without displaying any impairment in grades as an outcome (Gormley et al., 2015). This third form of psychological compensation, the acquisition of new skills, is equal to the effect of therapeutic interventions.

To overcome the difficulty of being precise with regard to which deficit a study about compensation in ADHD is referring to, the first adaptation to the framework is the introduction of the term *outcome* in order to clarify that the deficit and the outcome cannot be confounded (**Figure 1**). To draw sound conclusions, the deficit (independent variable), compensation (independent variable), and the outcome (dependent variable) need to be measured as three distinguishable variables. Outcome is usually measured on a higher level compared to deficit and compensation (**Figure 2**). A deficit in one study (e.g., ADHD symptoms) can be the outcome in another study.

### Awareness

The second prerequisite states that an individual is aware of the mismatch between his/her skills and the environmental demands; whereas the third prerequisite entails that the person decides to compensate. Only very few studies have asked participants with ADHD about how they actually evaluate their own performance although it would be of greatest importance to investigate whether individuals are aware of their own deficits (Owens et al., 2007). There are also only few studies asking participants with ADHD for concrete samples of compensation in their daily lives (Gormley et al., 2015). Furthermore, it is difficult to assess the awareness of the deficit and the conscious decision to compensate in neurobiological or neuropsychological studies.

Therefore, the second adaption is that a person with ADHD might not be aware of the deficit and might not have come to the decision to compensate deliberately. This adaptation seems of particular importance for studies at a neurobiological or psychological process level.

### Consequences

Distinguishing adaptive and maladaptive consequences of compensation is pivotal. Psychological compensation can prevent diagnosis of ADHD because it allows individuals to meet environmental demands although they still show deficits. If psychological compensation is adaptive and does not have negative consequences, this is highly desirable and it would be unnecessary to diagnose or treat the individual. However, if psychological compensation is maladaptive, as for example in cases where individuals invest extensive effort and time to meet educational demands and neglect social relationships, it can lead to an accumulation of other comorbid problems in addition to ADHD (Quinn, 2005). In this case psychological compensation prevents diagnosis, and thereby prevents access to treatment and the opportunity to learn about adaptive compensation.

Although consequences were already part of the original framework, they are of crucial importance for ADHD research. If the consequences are adaptive, the compensatory behavior could be promising for future interventions. However, if the consequences are maladaptive it is important to consider compensatory behaviors when diagnosing ADHD as they could disguise deficits in diagnostic procedures.

## Implications for Studies

Most studies of psychological compensation generate hypotheses instead of testing them. Results from cross-sectional studies comparing samples with and without ADHD not showing any differences or an even better performance in the ADHD sample can generate ideas of psychological compensation. However, to test hypotheses about psychological compensation in ADHD, we need to reveal that the deficit remains and that it really is compensation leading to nonexistent group differences. If compensation is successful, it will hide deficits at the next level (**Figure 2**). Therefore, a nonexistent group difference is a necessary but not sufficient condition to infer compensation. In order to draw conclusions about compensation, we need to measure deficit, compensation, and outcome independently and demonstrate that compensation leads to nonexistent group differences. Thus, we need to assess deficit and compensation as two separable independent variables and outcome as the dependent variable. In addition, we need to show that the deficit is stable (within-persons, i.e., comparing persons with ADHD at different points of time or under different conditions, or between-persons, i.e., comparing groups with ADHD and remitters) and that the compensation entails equal or better outcome despite the deficit.

Studies of psychological compensation in ADHD usually include only individuals with ADHD related deficits who are not able to meet environmental demands. However, psychological compensation can only be found in individuals with a deficit who are able to meet the environmental demands. Thus, by comparing samples with and without ADHD (as outcome) one will never be able to answer questions about compensation. Instead, compensation in ADHD can either be investigated in between-person designs of samples with the same deficit but various outcomes or longitudinally in within-person designs and naturally or experimentally varying environmental demands.

In summary, for the study of psychological compensation in ADHD we need a clear definition and operationalization of the deficit we are referring to, the compensation and the outcome. We need to include groups with ADHD that are not able to meet environmental demands, a group with ADHD that is able to meet environmental demands, and a group without ADHD that is capable of meeting environmental demands.

## Benefits of Studying Compensation in ADHD

Research on ADHD benefits from studying compensation, because compensation might hide a core deficit of ADHD. As a result, some individuals with ADHD do not receive a diagnosis resulting in underdiagnosis, especially in females (Quinn, 2005; Merkt and Gawrilow, 2014). Such an underdiagnosis might be particularly detrimental if the consequences of the psychological compensation are maladaptive, as for instance an obsessive–compulsive behavior to meet college demands (Merkt and Gawrilow, 2014). However, if consequences of psychological compensation are adaptive, knowledge about compensation could foster the development of interventions in ADHD.

## Conflict of Interest Statement

The authors declare that the research was conducted in the absence of any commercial or financial relationships that could be construed as a potential conflict of interest.

## References

[B1] American Psychiatric Association (2013). *Diagnostic and Statistical Manual of Mental Disorders*, 5th Edn. Washington, DC: American Psychiatric Association.

[B2] Bäckman L. Dixon R. A. (1992). Psychological compensation: a theoretical framework. *Psychol. Bull.* 112 259–283. 10.1037/0033-2909.112.2.259 1454895

[B3] Castellanos F. X. Tannock R. (2002). Neuroscience of attention-deficit/hyperactivity disorder: the search for endophenotypes. *Nat. Rev. Neurosci.* 3 617–628. 10.1038/nrn896 12154363

[B4] Gawrilow C. Petermann F. Schuchardt K. (2013). ADHD in preschool. *Kindheit Entwicklung* 22 189–192. 10.1026/0942-5403/a000116

[B5] Gormley M. J. Pinho T. Pollack B. Puzino K. Franklin M. K. Busch C. (2015). Impact of study skills and parent education on first-year GPA among college students with and without ADHD: a moderated mediation model. *J. Atten. Disord.* 10.1177/1087054715594422 [Epub ahead of print]. PMC4715995 26187415

[B6] Halperin J. M. Schulz K. P. (2006). Revisiting the role of the prefrontal cortex in the pathophysiology of attention-deficit/hyperactivity disorder. *Psychol. Bull.* 132 560–581. 10.1037/0033-2909.132.4.560 16822167

[B7] Merkt J. Gawrilow C. (2014). Health, dietary habits, and achievement motivation in college students with self-reported ADHD diagnosis. *J. Atten. Disord.* 10.1177/1087054714523127 [Epub ahead of print]. 24554297

[B8] Merkt J. Singmann H. Goossens-Merkt H. Kappes A. Wendt M. Gawrilow C. (2013). Flanker performance in female college students with ADHD: a diffusion model analysis. *Atten. Defic. Hyperact. Disord.* 5 321–341. 10.1007/s12402-013-0110-1 23712448

[B9] Millenet S. Hohmann S. Poustka L. Petermann F. Banaschewski T. (2013). Riscfactors and early symptoms of ADHD. *Kindheit Entwicklung* 22 201–208. 10.1026/0942-5403/a000118

[B10] Owens J. S. Goldfine M. E. Evangelista N. M. Hoza B. Kaiser N. M. (2007). A critical review of self-perceptions and the positive illusory bias in children with ADHD. *Clin. Child Fam. Psychol. Rev.* 10 335–351. 10.1007/s10567-007-0027-3 17902055

[B11] Quinn P. O. (2005). Treating adolescent girls and women with ADHD: gender-specific issues. *J. Clin. Psychol.* 61 579–587. 10.1002/jclp.20121 15723425

[B12] Schweitzer J. B. Faber T. L. Grafton S. T. Tune L. E. Hoffman J. M. Kilts C. D. (2000). Alterations in the functional anatomy of working memory in adult attention deficit hyperactivity disorder. *Am. J. Psychiatry* 157 278–280. 10.1176/appi.ajp.157.2.278 10671402

[B13] Sergeant J. (2005). Modeling attention-deficit/hyperactivity disorder: a critical appraisal of the cognitive-energetic model. *Biol. Psychiatry* 57 1248–1255. 10.1016/j.bps.2004.09.010 15949995

[B14] Söderlund G. B. W. Sikström S. Loftesnes J. M. Sonuga-Barke E. J. S. (2010). The effects of background white noise on memory performance in inattentive school children. *Behav. Brain Funct.* 6 1–10. 10.1186/1744-9081-6-55 20920224 PMC2955636

[B15] Sonuga-Barke E. J. S. (2003). The dual pathway model of AD/HD: an elaboration of neuro-developmental characteristics. *Biobehav. Rev.* 27 593–604. 10.1016/j.neubiorev.2003.08.005 14624804

[B16] Sonuga-Barke E. J. S. (2005). Causal models of attention-deficit/hyperactivity disorder: from common simple deficits to multiple developmental pathways. *Biol. Psychiatry* 57 1231–1238. 10.1016/j.biopsych.2004.09.008 15949993

[B17] Thapar A. Langley K. Asherson P. Gill M. (2007). Gene-environment interplay in attention-deficit hyperactivity disorder and the importance of a developmental perspective. *Br. J. Psychiatry* 190 1–3. 10.1192/bjp.bp.106.027003 17197648

[B18] World Health Organization (2009). *International Classification of Diseases and Related Health Problems (ICD-10)*, 10th revision. Genf: World Health Organization.

